# Relapsing insulin-induced lipoatrophy, cured by prolonged low-dose oral prednisone: a case report

**DOI:** 10.1186/1758-5996-3-33

**Published:** 2011-12-06

**Authors:** Ernst A Chantelau, Ruth Praetor, Jörg Praetor, Ludger W Poll

**Affiliations:** 1 Practice of Endocrinology and Diabetology, PD Dr.Kimmerle, Aachener Str.196, 40223 Düsseldorf/Germany; 2 Cäcilienstr.40, 48431 Rheine/Germany; 3 Department of Radiology, Berufsgenossenschaftliche Unfallklinik Duisburg GmbH, Großenbaumer Allee 250, 47249 Duisburg/Germany

## Abstract

**Introduction:**

Circumscript, progressing lipoatrophy at the insulin injection sites is an unexplained, however rare condition in diabetes mellitus.

**Case presentation:**

We report a case of severe localised lipoatrophy developing during insulin pump-treatment (continuous subcutaneous insulin infusion) with the insulin analogue lispro (Humalog^®^) in a woman with type-1 diabetes mellitus. After 11 months of progressing lipoatrophy at two spots on the abdomen, low-dose prednisone (5-10 mg) p.o. was given at breakfast for 8 months, whereby the atrophic lesions centripetally re-filled with subcutaneous fat tissue (confirmed by MRI) despite ongoing use of insulin lispro. However, 4 weeks after cessation of prednisone, lipoatrophy relapsed, but resolved after another 2 months of low-dose prednisone. No further relapse was noted during 12 months of follow-up on insulin-pump therapy with Humalog^®^.

**Conclusion:**

Consistent with an assumed inflammatory nature of the condition, low-dose oral prednisone appeared to have cured the lipoatrophic reaction in our patient. Our observation suggests a temporary intolerance of the subcutaneous fat tissue to insulin lispro (Humalog^®^), triggered by an unknown endogenous mechanism.

## Background

So-called insulin-induced lipoatrophy is a rare, albeit feared condition mostly in patients with type-1 diabetes mellitus. It leads to a total loss of subcutaneous fat tissue at the sites of insulin injections. Neither the pathogenesis of the condition is known, nor the apparent female preponderance [1-14]. The clinical appearance is notable for its lack of inflammatory features. Histopathological reports were inconsistent: while subcutaneous fat atrophy and fibrosis was common, immunological abnormalities were found only in one of the studies (see Table 1). Insulin-induced lipoatrophy has been reported with the use of various pharmaceutical preparations, long acting or short acting insulin preparations, insulin analogues or human insulin, and with subcutaneous injection or continuous subcutaneous infusion (via insulin pump) treatment alike. Injection site lipoatrophy was also observed with the use of the somatostatin-analogue octreotide [8].

**Table 1:** Histopathological findings in insulin-induced circumscript lipoatrophy, as reported in the literature.

Previous reports, author	**Reeves **[1]	**Jermendy **[5]	**Lopez **[10]	**Chantelau **[3]	present case
					
cases,n	5	1	4	1	
					
**Skin:**					
					
immunofluorescence staining*	positive	negative	not analysed	negative	not analysed
					
mononuclear cell infiltration	mild	none	not analysed	mild	not analysed
					
					
**Subcutaneous tissue:**					
					
fat tissue atrophy	present	absent?	present	present	present
					
focal fibrosis	absent?	absent?	present	present	present
					
lipoblast-like adipocytes	absent	present	absent	absent	absent
					
capillary proliferation	absent	present	absent	absent	absent
					
immunofluorescence staining*	not analysed	not analysed	negative	negative	not analysed
					
lymphocyte infiltration	absent	not analysed	mild	mild	not analysed
					
eosinophils infiltration	absent	not analysed	present	absent	not analysed
					
mast cells infiltration	absent	not analysed	present	absent	not analysed

*for IgG, IgA, IgM, C1, C3, and fibrin, or amyloid, respectively

## Case presentation

**Figure 1:**
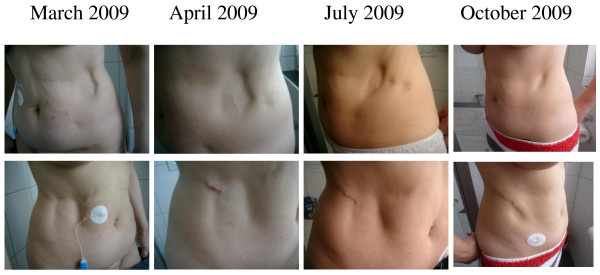
**Follow-up of lipoatrophic sites, before, during, and after prednisone therapy**. Series of photographs showing lipoatrophic areas on both sides of the umbilicus (upper panel: left side, lower panel: right side). Photographs as of March 2009 and April 2009 were taken before prednisone therapy. In April 2009, a biopsy was taken from the right side, which healed uneventfully, see lower panel. Low-dose oral prednisone was administered from the last week of June 2009 until March 2010, and from May 2010 until June 2010. To be continued on Figure 2.

**Figure 2:**
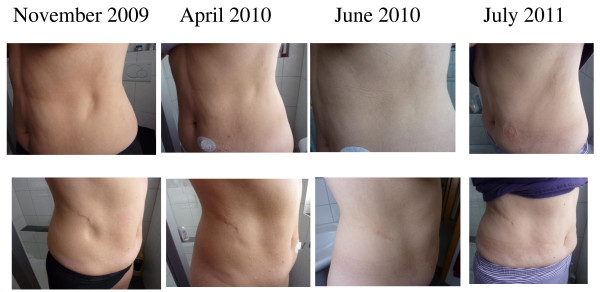
**Follow-up of lipoatrophic sites, before, during, and after prednisone therapy pt. 2**. Continuation from Figure 1. Low-dose oral prednisone was administered from the last week of June 2009 until March 2010, and from May 2010 until June 2010. The photographs as of July 2011 were taken 12 months after cessation of prednisone therapy.

**Figure 3:**
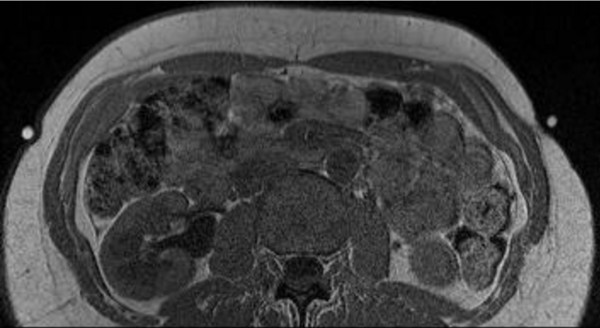
**MRI demonstrating re-grown subcutaneous fat tissue at sites of previous lipoatrophy**. Axial T1-weighted MRI sequence (1.5 Tesla Magnet, body surface coil, slice thickness 3 mm) acquired through the abdomen, March 2010. Markers are placed over the healed lipoatrophic area (left side), and over the biopsy scar (right side), respectively. Signal intensity and subcutaneous tissue structure indicate re-grown subcutaneous fat tissue at the healed area (left side; high signal intensity, identical to that of periumbilical subcutaneous fat tissue), and fibrous tissue at the biopsy area, respectively (right side; signal intensity is only half of that of subcutaneous fat tissue).

We present the case of a 53 year old caucasian woman (R.P., born 1956), who was diagnosed with type-1 diabetes mellitus at the age of 13 years. Since 1984 she was treated by continuous subcutaneous insulin infusion with a portable insulin pump using human regular insulin, without any adverse effects. Since 2004, insulin lispro (Humalog^®^, Eli Lilly Deutschland, Bad Homburg, Germany) was used for insulin pump treatment (currently used device: Accu-Check Spirit^®^, Roche, Burgdorf, Switzerland). There was no co-morbidity except for Hashimoto's thyreoiditis, requiring 100 μg levothyroxine once daily for substitution. There was no evidence for diabetic retinopathy, nephropathy or neuropathy, respectively. HbA1c (normal range 4-6% of total haemoglobin) was around 8%, body mass index was 23.8 kg/m^2^. In November 2007 and February 2008, vaccinations against hepatitis A and B were carried out with a vaccine containing traces of thiomersal (Twinrix^®^, GlaxoSmithKline, Dresden, Germany). Commencing in May 2008, a lipoatrophic defect developed at the catheter insertion site on the right side of the abdominal wall, and after changing to the opposite side, a second lesion developed there. The size of both lesions increased steadily. In March 2009 hollows of 6 × 7 cm had developed in the subcutaneous fat tissue (Figure 1). Routine blood chemistry was normal. Immunological serum markers (anti-insulin antibodies, as well as antibodies against mitochondria, c-ANCA, p-ANCA, and ANA IgG) were negative. Anti-TPO antibodies, however, were elevated (173 U/ml (normal < 60 U/ml)). In April 2009, a biopsy was obtained from the margin of the lipoatrophic area at the right lateral abdominal wall (Figure 1, lower panel). Routine histopathologic examination showed atrophic fat tissue (lipoatrophy); unfortunately, more detailed histochemical investigations were not performed. Assuming a potential adverse effect of Humalog^®^, the insulin preparation was changed-over to rapid-acting genetically engineered human insulin (Actrapid^®^, NovoNordisk, Mainz, Germany), without obvious benefit. In consideration of an undefined immunologic pathogenesis, low-dose immunosuppressive treatment with 10 mg prednisone once daily was commenced on June 23, 2009 (with adaptations of the insulin dosage, as appropriate, to maintain HbA1c between 7 and 8%). As an immediate effect, no new lipoatrophic hollows developed at the catheter insertion sites in use. In August 2009, the existing hollows started to re-fill, beginning at the margins (Figure 1, 2). In September 2009, the daily prednisone dosage was reduced to 5 mg, and further to 2.5 mg in October 2009. In December 2009, the patient decided to switch back from human insulin (Actrapid^®^) to insulin lispro (Humalog^®^), while the lipoatrophic hollows continued to re-fill with subcutaneous fat tissue rather than fibrous scar tissue (confirmed by MRI, see Figure 3). In March 2010, when the lipoatrophy was almost cured, prednisone was discontinued. The patient was advised to use the previously lipoatrophic areas again for catheter insertion. However, about 4 weeks later, prednisone had to be resumed because lipoatrophy had relapsed. By June 2010, after another 2 months of prednisone therapy, the lipoatrophic areas were cured again (Figure 2) and prednisone was tapered off. During the following 12 months on unchanged insulin-pump therapy using insulin lispro, no relapse of the lipoatrophy was noted (Figure 2).

However, 3 months after cessation of prednisone treatment, in both hands cheiroarthropathy developed with thickened flexor digitorum tendons and edema of the tendon sheaths, as evidenced by MRI [15]. Rheumatoid arthritis was ruled out on laboratory and imaging studies. The condition was markedly alleviated by physiotherapy.

## Discussion

Our present observation in an adult patient is the first to show an acutely relapsing circumscript lipoatrophy at the sites of subcutaneous infusion of insulin lispro, which fully resolved by low-dose oral corticosteroid treatment. In a previous patient of ours, whose lipoatrophies seemingly stopped after changing from injections to insulin pump treatment [3], a relapse occurred after 5 years of insulin pump treatment and resolved during oral corticosteroid treatment (given to treat newly diagnosed arteriitis temporalis [E.A. Chantelau, unpublished observation]).

In contrast to adult patients, in whom insulin lipoatrophy rarely resolves spontaneously [2,7,10,11,14], insulin lipoatrophy sometimes resolved spontaneously in children [5,6,12,16]. Hence, treatment of the condition is particularly warranted for adults. Changing the insulin brand, or changing from injections to continuous subcutaneous insulin infusion [3,11] seemed to be equally ineffective. Empirical treatment based on topical corticosteroid injections (injection of dexamethasone into the lipoatrophic lesions) had resulted in return of subcutaneous fat tissue in an adult [17,18] and in a child [9], but not in an infant [12]. Recently, topical disodium cromoglycate ointment was found effective in lipoatrophy of short duration by Lopez et al., who also reported that a long-standing chronic lipoatrophy was unresponsive to either topical disodium cromoglycate ointment or corticosteroid injections [10].

The present case report adds to the evidence, that a subclinical, subacute inflammatory reaction may be involved in the pathogenesis of insulin-induced lipoatrophy, since the destructive process may be suppressed and even reversed by administration of anti-inflammatory compounds (e.g. corticosteroids, disodium cromoglycate ointment). This process is different from the local inflammatory reaction that regularly occurs in the subcutaneous fat tissue after injection of insulin, insulin analogues or other compounds [1,10,19,20]. However, the underlying mechanisms of injection-induced subcutaneous lipoatrophy remain to be determined. A personal susceptibility might be involved, as has been suggested by Atmaca et al. [8], which might be transitory, as our case seems to indicate. The trigger of such a transitory intolerance remains unknown, as well as the female preponderance of the condition.

## Conclusion

In conclusion, the case of our patient supports the use of prolonged low-dose corticosteroid treatment to cure progressive insulin-induced lipoatrophy. A careful adaptation of the insulin dose is mandatory in order to avoid corticosteroid-induced hyperglycaemia.

## Consent

Written informed consent was obtained from the patient for publication of this case report and accompanying images. A copy of the written consent is available for review by the Editor-in-Chief of this journal.

## Competing interests

The authors declare that they have no competing interests.

## Authors' contributions

EC conceived the idea, compiled the data and drafted the paper. RP and JP contributed patient data and the photographs. LWP provided the MR imaging analysis and participated in writing the paper. All authors read and approved the final manuscript.
